# Gastrointestinal Symptoms in Obesity Therapy: Mechanisms, Epidemiology, and Management Strategies

**DOI:** 10.3390/biomedicines13102362

**Published:** 2025-09-26

**Authors:** Tomasz Witaszek, Aleksander Biesiada, Joanna Iskra-Trifunović, Mateusz Babicki, Agnieszka Mastalerz-Migas, Karolina Kłoda

**Affiliations:** 1 Independent Researcher, ul. Józefińska 33/8, 30-529 Kraków, Poland; 2 Polish Society of Family Medicine, ul. Syrokomli 1, 51-141 Wrocław, Poland; alek.biesiada@gmail.com (A.B.); wikarla@gazeta.pl (K.K.); 3 Independent Researcher, ul. Wspaniała 15, 72-006 Mierzyn, Poland; iskra.joanna@gmail.com; 4 Department of Family Medicine, Faculty of Medicine, Wroclaw Medical University, 50-367 Wrocław, Poland; ma.babicki@gmail.com (M.B.); agnieszka.mastalerz-migas@umw.edu.pl (A.M.-M.)

**Keywords:** obesity, obesity management, anti-obesity medications, gastrointestinal adverse effects

## Abstract

Obesity management, whether lifestyle-based, pharmacological, or surgical, is frequently associated with gastrointestinal adverse effects (GI AEs) that may impact treatment adherence and patient quality of life. With the increasing use of incretin-based anti-obesity medications (AOMs), they have gained particular clinical relevance. This review aims to explore current evidence on the prevalence, underlying mechanisms, and management strategies for GI AEs associated with obesity therapies, with a particular focus on nausea, diarrhea, constipation, gastroesophageal reflux and cholelithiasis. A search of PubMed and Scopus was conducted for articles published between 2006 and 2025. Eligible studies included randomized controlled trials, observational studies, and narrative or systematic reviews reporting on GI AEs in the context of obesity treatments, especially those involving incretin-based AOMs. Clinical trial data on AOMs indicate that GI AEs are reported in 65–84% of patients treated with liraglutide, semaglutide or tirzepatide, with the most common being nausea and diarrhea. These symptoms are primarily attributed to altered gastric motility and hormone-mediated changes in appetite signaling. Preventive strategies such as slow dose titration, dietary counseling, and supportive medications are commonly recommended to support tolerability and treatment continuation. GI AEs remain a common and often underestimated barrier to effective obesity management. Early recognition and structured management are essential to long-term success. Clinicians should incorporate anticipatory counseling and shared decision-making at treatment initiation to set realistic expectations, optimize tolerability, and support adherence.

## 1. Introduction

Obesity is a recognized chronic condition [1], the pathophysiology of which involves complex interactions between genetic, environmental, and behavioral factors. This leads to excessive fat accumulation that presents health risks far beyond one’s appearance [2]. 60% of citizens in the area of Europe are either overweight or living with obesity. That number has nearly tripled since 1975 and it is now considered an epidemic with severe health, social, and economic consequences [3].

Obesity management has evolved in recent years, expanding beyond lifestyle modifications and bariatric procedures. Novel incretin-based therapies have provided a non-invasive yet highly effective option for patients [4]. While these interventions have proven successful in promoting weight reduction and metabolic improvement, they may also cause adverse effects [5].

Gastrointestinal adverse effects (GI AEs) commonly occur both as comorbidities of obesity itself and as side effects of obesity treatment regimens. An increase in BMI is associated with more frequent gastrointestinal (GI) symptoms such as upper abdominal pain, gastroesophageal reflux (GERD), diarrhea, heartburn, vomiting, and incomplete bowel movements [6]. Similarly, the most frequent side effects of anti-obesity medications (AOMs) are also GI in nature [5]. Although usually transient and non-severe, these effects can still impact patients’ quality of life and adherence to treatment, and can be a reason for discontinuing the treatment. The gut–brain axis plays a critical role in energy homeostasis and appetite regulation, making it both a target for therapeutic intervention and a source of treatment-related adverse effects [7]. As most physicians now encounter patients receiving AOMs, understanding the mechanisms behind these symptoms is crucial for clinical management and patient education.

This review aims to comprehensively examine GI symptoms associated with obesity and its treatments, with a particular focus on those caused by incretin-based therapies. It explores underlying mechanisms, epidemiological patterns, and evidence-based management strategies. Despite the clinical relevance of GI AEs, there is currently no clear consensus on optimal strategies for their prevention and management. Existing guidelines are often based on expert opinion or extrapolated from limited data. Therefore, the goal is to provide clinicians with practical guidance for optimizing obesity treatment outcomes while minimizing GI AEs.

## 2. Methods

This narrative review is based on a literature search conducted in PubMed and Scopus for articles published between 2006 and 2025. The last search was conducted on 8 September 2025. Search terms included combinations of obesity, anti-obesity medication, GLP-1, GIP and gastrointestinal adverse effects. Priority was given to randomized controlled trials, meta-analyses, and large observational studies, while relevant narrative and systematic reviews were also included to provide additional context. Articles not published in English, conference abstracts, and studies without original data were excluded. The selection aimed to provide a comprehensive yet focused overview of GI AEs associated with pharmacological treatment of obesity.

## 3. Symptoms Associated with Different Intervention

### 3.1. Diet and Physical Activity

It is worth noting that patients who are not receiving pharmacological treatment may still be at risk of developing GI symptoms. A healthier diet—characterized by lower fat content and higher intake of fruits and dietary fiber is generally associated with a reduced incidence of GI symptoms in people living with obesity [8]. However, specific dietary patterns and food components can both trigger and alleviate various GI complaints. For instance, high-fiber diets, although typically beneficial for digestive health, may cause significant discomfort when introduced too rapidly or consumed in excessive amounts. Common adverse effects include bloating, flatulence, constipation, abdominal discomfort, and pain [9]. Conversely, high-fat diets—such as ketogenic or paleo diets—may lead to nausea, constipation and other GI disturbances in some individuals [10]. Additionally, physical activity promotes gut motility and supports overall digestive health, with evidence from weight-loss program participants showing that higher activity levels are associated with fewer GI AEs [11].

Excess body weight itself is an established risk factor for gallstone formation. Especially rapid weight loss—often observed during very-low-calorie diets—may further promote the development of gallstones or exacerbate symptoms of preexisting cholelithiasis [12]. This is due to several mechanisms, including hepatic overproduction of cholesterol—leading to bile supersaturation—and reduced gallbladder motility. Together, these factors promote cholesterol crystal formation and gallstone development, with the risk rising proportionally to body weight [13]. Although the absolute risk of symptomatic gallstones requiring hospitalization or cholecystectomy remains low, it has been shown to be approximately three times higher in individuals following very-low-calorie diets compared to those on low-calorie diets during a one-year weight reduction program [14]. Gradual weight loss, in contrast, may help mitigate this risk [12]. Recently, this issue has gained renewed attention with the rise of bariatric surgery and incretin-based therapies, which can accelerate weight loss and increase gallstone risk—a topic discussed further in this review.

### 3.2. GI Symptoms Associated with the Use of Incretin-Based Therapies

GI AEs are the most frequently reported side effects of GLP-1 receptor agnonists (GLP-1RA) and dual GIP/GLP-1 receptor agonists (GIP/GLP-1RA), occurring regardless of the pharmacokinetic profile (short- or long-acting) or route of administration (subcutaneous or oral). Clinical trial data on AOMs indicate that GI AEs are reported in 65–84% of patients treated with liraglutide, semaglutide or tirzepatide [15,16,17,18,19,20,21,22,23,24,25,26,27]. The most common GI AEs across all AOMs are nausea and diarrhea. These events are typically mild to moderate in intensity, transient, and most often emerge during the dose-escalation phase, subsiding shortly after reaching the maintenance dose [28]. Comparable real-world data are scarce, as mild to moderate GI symptoms do not necessarily lead to healthcare contacts or registration in medical records or databases. In a community pharmacy-based survey of semaglutide users, six in 10 users reported having experienced side effects, most commonly nausea (35%) and constipation (29%) [29].

In head-to-head trials, treatment discontinuations due to GI AEs were more frequent with liraglutide than with semaglutide—12.6% (95% CI, 7.9–19.5) vs. 3.2% (95% CI, 1.2–7.9), with approximately half of liraglutide discontinuations due to GI AEs, most of which occurred during dose escalation [17]. Similarly, in the SURMOUNT-5 trial comparing semaglutide with tirzepatide, GI AEs were the leading cause of treatment discontinuation and occurred more often with semaglutide than with tirzepatide—5.6% (95% CI, 3.7–8.4) vs. 2.7% (95% CI, 1.5–4.9) [23].

The mechanisms behind these symptoms are likely multifactorial [30]. Central mechanisms, such as activation of GLP-1 receptors (GLP-1R) in the area postrema, are thought to contribute to nausea. Peripheral effects including delayed gastric emptying, changes in motility, and altered transit time may also play a role. The effect on gastric emptying usually weakens over time with chronic exposure in long-lasting agents due to tachyphylaxis, suggesting that persistent GI symptoms cannot be explained by this mechanism alone [30]. Tirzepatide appears to be better tolerated than semaglutide, which may relate to its dual GLP-1R and GIP receptor (GIPR) action [23]. While GIP has no meaningful effect on gastric emptying, emerging data suggest that GIPR activation may have anti-emetic properties, which could mitigate GI AEs occurrence [31].

A comparative summary of key AOM clinical trials is provided in Table 1. Results are reported as the percentage of patients in the treatment cohort who experienced the adverse event at least once during the study period.

### 3.3. GI Symptoms Associated with the Use of Other AOMs

The combination of naltrexone and bupropion is associated with GI AEs primarily attributable to the pharmacological properties of each component. The most frequently reported symptoms include nausea, constipation, vomiting, and diarrhea [32]. These events are typically mild to moderate in intensity, occur early in the course of treatment—particularly during dose titration—and rarely lead to treatment discontinuation. Phentermine/topiramate therapy is also associated with GI complaints, most commonly dysgeusia (altered taste), dry mouth, and constipation [33]. Orlistat, a lipase inhibitor that reduces fat absorption in the intestine, is frequently linked to GI AEs such as steatorrhea, diarrhea, abdominal discomfort, and oily spotting [34]. These effects are often considered significant by patients and have contributed to the declining use of orlistat in current obesity pharmacotherapy.

### 3.4. GI Symptoms Associated with Bariatric Surgery

Bariatric surgery is a well-established and effective treatment for obesity. Several surgical procedures are currently performed, each associated with varying degrees of efficacy and risk, with sleeve gastrectomy and Roux-en-Y gastric bypass being the most common [35]. Among the most significant complications following bariatric surgery are GI AEs. GERD may occur, particularly after sleeve gastrectomy. Due to the reduced gastric volume and increased intraluminal pressure, reflux symptoms may worsen. Dumping syndrome is another common complication. It occurs when food, especially meals rich in sugar or fat, passes too rapidly from the stomach into the small intestine, overwhelming the digestive system. Symptoms include nausea, abdominal cramps, diarrhea, dizziness, and sweating. Early dumping syndrome develops within 30 min of eating as a consequence of accelerated gastric emptying, whereas late dumping syndrome appears 1–3 h postprandially and is typically linked to excessive insulin release in response to high glucose loads in the small intestine. Nausea and vomiting are also frequently reported after bariatric procedures and may occur in both early and late postoperative phases, depending on the underlying etiology. In addition, gallstone formation is a well-documented complication, particularly in patients experiencing rapid and substantial weight loss [36,37].

## 4. Prevention and Management of GI AEs During AOMs Use

As GI AEs are among the most common side effects of AOMs, prevention and management strategies are crucial for therapeutic success and patient comfort. Although usually mild to moderate in severity and transient in duration, these symptoms can negatively impact treatment adherence. They can be better managed by both non-pharmacological and pharmacological interventions. Although these symptoms can occur in any patient, identifying individuals at higher risk is essential for early recognition and personalized management. Key vulnerable groups include frail individuals and those with pre-existing GI disorders or gallbladder disease [28].

### 4.1. Lifestyle Modifications

Clinical guidelines and expert consensus emphasize the importance of behavioral and dietary interventions as first-line strategies to mitigate GI symptoms [28,38]. Recommended non-pharmacologic measures include:

Meal size and timing

- Eating slowly and chewing food thoroughly.
- Consuming food only in response to physiological hunger cues.
- Reducing portion sizes.
- Increasing meal frequency while decreasing portion size.
- Avoiding food intake close to bedtime.
- Discontinuing eating right after achieving satiety.

Food composition

- Preferring easily digestible, low-fat meals.
- Choose foods with lower viscosity, lower glycemic index, and higher water content to promote gastric emptying,
- Using low-fat cooking methods such as boiling, baking, or grilling.
- Limit alcohol intake, as it can exacerbate nausea and GERD.

Hydration

- Maintaining proper hydration by drinking small sips regularly,
- Avoid large fluid volumes in a short time to prevent gastric distension and dehydration, especially during periods of nausea, vomiting, or diarrhea.
- Avoiding the use of straws to minimize aerophagia.

Activity and posture

- Avoiding lying down immediately after meals.
- Refraining from intense physical activity shortly after eating.

It is important to thoroughly educate patients about the most common adverse effects and prophylactic strategies before initiating AOMs. Counseling in primary care plays a key role, and ensuring access to dietetic support can further enhance patient tolerance, particularly during dose escalation. A practical patient handout summarizing these strategies may also be helpful to reinforce guidance between visits.

### 4.2. Minimizing GI AEs: Clinical Management Strategies

Before attributing symptoms to AOMs, clinicians should evaluate for alternative or contributing causes. This includes ruling out GERD, GI infections, or adverse effects from concomitant medications such as metformin. Supportive interventions may help reduce symptom burden and improve adherence [28,38]:

○ Ensuring sufficient hydration, particularly in the presence of vomiting.
○ Providing individualized counseling on lifestyle and dietary strategies.
○ Considering short-term pharmacologic interventions, such as anti-emetics, prokinetic agents or proton pump inhibitors, when clinically appropriate. Long-term use of symptomatic treatments without addressing underlying causes should be avoided.

**Figure 1:**
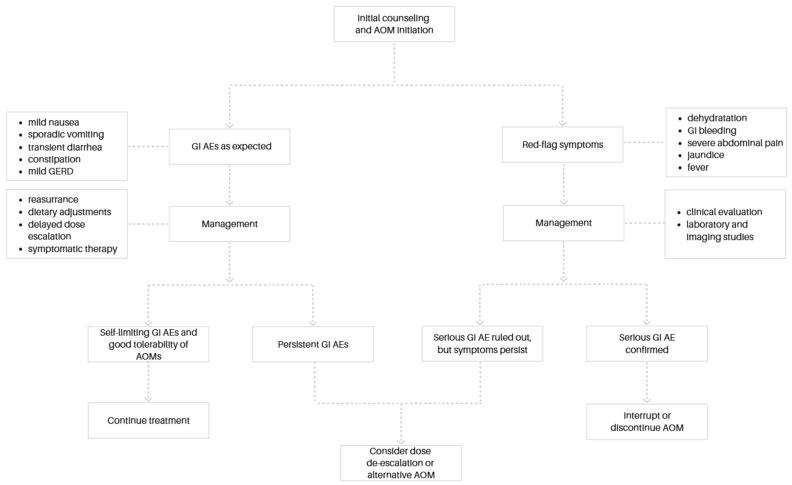
Algorithm for the prevention and management of GI AEs during AOM therapy.

In cases of poor tolerability, dose adjustment may be necessary. Slowing the rate of dose escalation or temporarily reducing the dose may alleviate symptoms. Following symptom resolution, re-escalation can be attempted at a slower pace to improve tolerance. Persistent GI adverse events, despite dose modification, may warrant a change in the pharmacological agent. Switching to a different GLP-1RA or transitioning to an alternative class of AOMs should be considered. If symptoms remain severe or unresponsive to supportive measures and medication adjustment, treatment discontinuation may be required. In such cases, alternative therapeutic strategies should be pursued to maintain progress in obesity management. An overview of practical strategies for the management of GI AEs is presented in Figure 1.

## 5. Management of Specific GI AEs

Certain symptoms may require tailored management strategies. Here, we present current knowledge on the recognition and management of the most common GI AEs observed in clinical practice.

### 5.1. Nausea and Vomiting

Nausea is the most common GI AE after initiation of GLP-1RA in people with obesity, with the highest rates reported in STEP-8 study for semaglutide 2.4 mg–61.1% (95% CI 52.4 to 69.2) [17]. It typically occurs within the first 4–5 weeks of treatment and tends to resolve within about a week [28].

To help relieve symptoms, patients may be advised to eat bland, soothing foods about 30 min after dosing—such as crackers, apples, peppermint, or ginger (e.g., ginger tea). Avoiding strong smells and eating smaller, more frequent meals can also be beneficial [28,38].

Scientific data on the use of anti-emetics alongside AOMs remains limited. However, we have some data from other incretin medications. In a retrospective analysis on healthy volunteers, patients premedicated with anti-emetics—specifically ondansetron or metoclopramide—experienced significantly lower rates of nausea and vomiting (16.7% and 6.7%, respectively) compared to those who received no premedication during treatment with exenatide [39]. It is important to note, as exenatide is a short-acting agent, it does not have the same tachyphylaxis risks as long-acting AOMs. Clinical recommendations suggest the use of anti-emetics when symptoms persist despite dose adjustments, after ruling out contraindications, prescribed for the shortest possible duration, at the lowest effective dose, and with careful patient monitoring [28]. Domperidone may be preferred over metoclopramide in older adults because it lacks extrapyramidal risks, but its use is limited in some jurisdictions owing to concerns about QT prolongation [40]. Metoclopramide should be reserved for short-term use because of the risk of tardive dyskinesia [41].

Although not specifically investigated in the context of AOM use, prokinetic agents such as cinitapride and itopride may offer therapeutic potential, particularly when first-line options are unavailable. Both have demonstrated efficacy in reducing postprandial fullness and early satiety in patients with functional dyspepsia [42,43]. It is important to emphasize, however, that the GI effects of GLP-1RA and GIP/GLP-1RA extend beyond delayed gastric emptying and involve complex peripheral and central neurohormonal mechanisms. Thus, use of prokinetic agents does not affect their course of action.

### 5.2. Diarrhea

Diarrhea has been reported in 18–36% of patients treated with incretin based AOMs [17,20]. The highest incidence typically observed during the first four weeks of therapy and symptoms tend to decrease significantly over time [28].

Recommendations include maintaining adequate hydration while avoiding sports drinks designed for physical activity. Patients should also avoid dairy products, fruit juices, laxative foods, coffee, alcohol, carbonated beverages, extremely hot or cold meals, and products containing sugar alcohols (e.g., sorbitol, mannitol, xylitol, maltitol). A temporary reduction in dietary fiber intake may also be beneficial [28,38].

If non-pharmacological measures are insufficient, short-term use of loperamide may be considered [28,44]. While direct evidence in the context of incretin medication is limited, loperamide has shown benefits in improving stool consistency and continence in individuals with obesity treated with orlistat [45].

### 5.3. Constipation

Constipation is a frequent GI AE of incretin medications, with STEP-8 reporting an incidence of 39% (95% CI 30.8–47.6) among participants treated with semaglutide 2.4 mg [17]. Compared with other GI AEs, constipation tends to persist longer; in STEP 1–3, the average duration was 47 days in people with obesity, with prevalence plateauing around the 10th week after initiation of treatment [18,19,20].

Management strategies include ensuring adequate hydration and gradual increases in dietary fiber intake, while cautioning against excessive fiber restriction, which may worsen symptoms in the long-term. Regular physical activity and a healthy, balanced diet are encouraged. Patients may also temporarily reduce intake of very high-protein or high-fat foods if constipation worsens [28,38].

If lifestyle measures are insufficient, osmotic laxatives such as macrogols or lactulose are preferred as first-line agents. Stimulant laxatives may be used short-term if osmotic agents are ineffective or poorly tolerated. Although no randomized controlled trials have evaluated these interventions specifically in the context of AOM therapy, their efficacy in managing functional constipation is well established [46].

### 5.4. GERD

The relationship between obesity and GERD is well established, with studies showing substantial symptom improvement following weight reduction [47]. By contrast, the pathophysiology of GERD in patients receiving AOMs is less clearly defined and may involve delayed gastric emptying, gastric distension, and relaxation of the lower esophageal sphincter. Most studies examining the relationship between GLP-1RA–induced gastric emptying delay and GI symptoms in obesity have found only weak or no correlations [48]. In contrast, a recent retrospective study of subcutaneous semaglutide use (primarily in patients with obesity without T2D) reported a significant association between GI symptoms, such as dyspepsia, and measures of gastric emptying [49]. The development of tachyphylaxis to the gastric emptying effect over time may partly explain why the risk of GERD decreases with longer treatment duration [30].

However, if GERD symptoms persist, in addition to non-pharmacological measures, short-term use of proton pump inhibitors (PPIs) may be considered to alleviate symptoms before further dose escalation [28]. While PPIs remain the core of medical treatment for GERD, acid-neutralizing agents and mucosal protective agents may also be used as adjunctive therapies, in accordance with current clinical guidelines [50]. The combination therapy of PPIs with prokinetics is being continuously investigated and is gaining importance in recent guidelines [51]. This approach has demonstrated efficacy and is well tolerated by patients [52].

### 5.5. Cholelithiasis

Pancreatobiliary complications have been documented, although they are considered rare [53]. Meta-analyses indicate that GLP-1RA may increase the risk of gallbladder and biliary tract disorders. This risk appears to be dose-dependent and more pronounced with longer treatment durations, particularly in the context of weight loss therapy. Potential mechanisms include both weight-loss–mediated effects (increased gallstone formation during rapid weight reduction) and drug-related effects such as altered biliary secretion or impaired gallbladder motility [54].

Avoiding rapid weight loss and very low-calorie diets may help reduce the risk, while higher-fat diets have also been shown to lower gallstone formation compared to low-fat diets [12,13,55].

Evidence from a meta-analysis of randomized controlled trials suggests that ursodeoxycholic acid (UDCA) significantly reduces the risk of gallstone formation compared to control interventions during weight loss. This protective effect is especially evident in patients following dietary weight loss interventions, though it is also relevant in post-bariatric surgery patients. UDCA additionally lowers the likelihood of requiring cholecystectomy due to symptomatic gallstones. In patients after bariatric surgery, UDCA at a dose of 500–600 mg daily for six months reduces gallstone incidence from 38% to 8%, and such preventive treatment is recommended [56]. Joint guidelines from the European Society for Clinical Nutrition and Metabolism and United European Gastroenterology on obesity care in patients with GI and liver diseases recommend UDCA in patients undergoing weight reduction interventions such as lifestyle and dietary changes, endoscopic procedures, and surgery [57]. They do not specifically mention AOMs. Similarly, the European Association for the Study of the Liver Clinical Practice Guidelines recommend treatment with UDCA until body weight is stabilized—but again, this is in the context of very low-calorie diets and bariatric surgery, not pharmacological treatment [58]. A more recent report suggests doses of ≥500 mg per day, but again focusing on the metabolic surgery induced weight loss setting [59].

With the introduction of highly effective AOMs such as tirzepatide, which can achieve mean weight loss exceeding 20% of initial body weight [23], the potential role of UDCA prophylaxis needs to be reconsidered. However, as the clinical use of these agents is relatively recent, evidence for prophylaxis in the context of pharmacotherapy-induced weight loss is not yet established.

## 6. Conclusions

AOMs, particularly novel incretin-based agents, can substantially enhance weight loss outcomes. However, they can sometimes be introduced without sufficient patient education and support. Patients should be informed that GI AEs are common, typically mild to moderate in intensity, and usually transient. Shared decision-making and anticipatory counseling at treatment initiation and dose escalation are essential to prepare patients for these possibilities and to improve adherence. Comprehensive obesity management involves not only lifestyle counseling and pharmacologic intervention, but also appropriate dose adjustments, proactive management of common GI AEs, and symptomatic treatment when adverse effects persist. Importantly, variability in access to care, monitoring capacity, and the burden of comorbidities means that tolerability and management strategies must be individualized. Given that GI symptoms often coexist—such as nausea with GERD or constipation—combination strategies targeting multiple symptoms may be necessary to optimize patient comfort and sustain long-term treatment compliance.

## Figures and Tables

**Table 1:** Frequency of GI AEs in clinical trials with incretin-based AOMs in people with obesity.

AOM	Program	GI AEs	Nausea	Vomiting	Diarrhea	Constipation	Special Considerations
Liraglutide 3 mg	SCALE Obesity and Prediabetes [15]		40	16	21	20	
	SCALE Diabetes [16]	65	33	16	26	16	adults with overweight or obesity and type 2 diabetes (T2D)
	STEP 8 [17]	83	59	21	18	32	
Semaglutide 2.4 mg	STEP 1 [18]	74	44	25	32	23	
	STEP 2 [19]	64	34	22	21	17	adults with overweight or obesity and T2D
	STEP 3 [20]	83	58	27	36	37	
	STEP 4 [21]	71	47	16	24	22	GI AEs during the run-in period (weeks 0–20)
	STEP 5 [22]	82	53	30	35	31	
	STEP 8 [17]	84	61	25	28	39	
	SURMOUNT-5 [23]		44	21	23	29	
Tirzepatide 15 mg	SURMOUNT-1 [24]		31	12	23	12	
	SURMOUNT-2 [25]		22	13	22	9	adults with overweight or obesity and T2D
	SURMOUNT-3 [26]		40	18	31	23	
	SURMOUNT-4 [27]		35	16	21	21	GI AEs during the run-in period (weeks 0–36)
	SURMOUNT-5 [23]		44	15	24	27	

## Data Availability

Not applicable.
